# Identifying and understanding the care pathway of patients with atrial fibrillation in Brazil and the impact of the COVID-19 pandemic: A mixed-methods study

**DOI:** 10.1371/journal.pone.0292463

**Published:** 2023-10-12

**Authors:** Alessandra C. Goulart, Ana C. Varella, Tiffany E. Gooden, Gregory Y. H. Lip, Kate Jolly, G. Neil Thomas, Paulo A. Lotufo, Sheila Greenfield, Rodrigo D. Olmos, Isabela M. Bensenor, Semira Manaseki-Holland, on behalf of the NIHR Global Health Research Group on Atrial Fibrillation Management

**Affiliations:** 1 Center for Clinical and Epidemiologic Research and Division of Internal Medicine, University Hospital, University of São Paulo, São Paulo, Brazil; 2 Institute of Applied Health Research, University of Birmingham, Birmingham, United Kingdom; 3 Liverpool Centre for Cardiovascular Science at University of Liverpool, Liverpool John Moores University and Liverpool Heart & Chest Hospital, Liverpool, United Kingdom; 4 Department of Clinical Medicine, Aalborg University, Aalborg, Denmark; 5 Medical School, Universidade de São Paulo, São Paulo, Brazil; Federal University of Santa Catarina: Universidade Federal de Santa Catarina, BRAZIL

## Abstract

**Background:**

Atrial fibrillation (AF) is a major risk factor for stroke. To enable improvements to AF diagnosis and follow-up care, understanding current patient pathways and barriers to optimal care are essential. We investigated the patient care pathways and their drivers, and the impact of the COVID-19 pandemic on patient pathways in a middle-income country setting, Brazil.

**Methods:**

This mixed-methods study in São Paulo, included adults (≥18y) with AF from 13 primary/secondary healthcare facilities. Surveys using baseline, follow-up (administered ≥two months after baseline) and COVID-19 questionnaires (quantitative), and three focus group discussions (FGDs) were conducted. Minimum sample size for the quantitative component was 236 and we aimed to reach saturation with at least three FGDs for the qualitative component. Descriptive statistics were used for quantitative data and a content analysis was used for qualitative data to identify themes related to AF diagnosis and follow-up care.

**Results:**

267 participants completed the baseline questionnaire: 25% were diagnosed in primary care, 65% in an emergency or inpatient department. At follow-up (n = 259), 31% visited more than one facility for AF care, and 7% had no follow-up. Intervals between international normalised ratio (INR) tests were increased during the pandemic, and the number of healthcare visits and availability of medication were reduced. Seventeen patients participated in three FGDs and revealed that AF diagnosis often occurred following a medical emergency and patients often delay care-seeking due to misconceptions about AF symptoms. Long waiting times, doctor/patient interactions and health system factors, such as doctor availability and the referral system, influence where participants visited for follow-up care.

**Conclusions:**

Lack of public awareness and underdeveloped primary healthcare lead to delayed diagnosis, which impacts clinical outcomes and excess patient and healthcare system costs. Health system, care-provider, and pandemic factors disrupt timely and effective continuity of care.

## Introduction

In 2017, globally, more than 37 million individuals had a diagnosis of atrial fibrillation (AF), an increase of 33% since 1997 [1]. However, AF prevalence increased the most in low- and middle-income countries (LMICs) [1], in part due to changing lifestyles leading to non-communicable diseases (NCDs) and a rise in life expectancy in LMICs thus an increase in a key risk factor for AF, older age [2]. Around 20% of ischaemic strokes are due to AF and this increases with age [3]; however, oral anticoagulants (OACs), such as warfarin, can reduce stroke by 64% [4]. Warfarin is globally common and extensively available in most LMICs, but requires careful monitoring to reduce risk of thrombosis and haemorrhage through routine INR (international normalised ratio) tests to ensure effective therapeutic range [5]. As the burden of AF and other NCDs increase globally, it is imperative to understand the patient pathway and gaps in care for AF, particularly in LMICs where resources are limited and thus access and quality often differ from high-income countries (HIC).

The AF Better Care (ABC) pathway [6] has been implemented and effective in reducing MACE (major adverse cardiovascular events) and mortality in many HICs but evidence is limited in LMICs. The ABC pathway proposes the following: (A) avoid stroke with anticoagulants; (B) better symptom management, with patient‐centred symptom-directed decisions on rate or rhythm control; and (C) cardiovascular and comorbidity risk optimisation, including lifestyle changes [6]. Identifying opportunities for improvement within the current AF care pathways will enable the development of interventions, service improvements and policy changes to enhance the efficiency and effectiveness of AF care and optimise delivery of the ABC pathway. It is also crucial to identify where the pathways are vulnerable to disruptions during unexpected events, such as the COVID-19 pandemic, to understand where the pathway needs strengthening to ensure the same level of continuity of care and patient safety can be maintained and poor clinical outcomes can be avoided in future crises. Lessons learnt from one country setting can apply to other countries since for example many patterns of barriers or facilitators to optimal care are shared across health systems and patient populations in LMICs.

As part of a multi-country AF research group, we aimed to determine the AF care pathway in a large middle-income country, Brazil, where stroke and AF pose a significant burden of disease (2.4% prevalence of AF among the older population [7] and 17% of strokes are AF-related [8] in our study site). We investigated where AF diagnosis, follow-up care and management occur, and the contextual factors driving patients’ pathway of AF care. A secondary aim, which became feasible opportunistically due to occurrence of the COVID-19 pandemic mid-way through the study, was to identify the impact of the pandemic on the AF care pathway.

## Materials and methods

### Population and study design

This study was part of a multi-country mixed-methods study design [9, 10]. A mixed-methods design was chosen to allow for contextual drivers to be identified (qualitative component) for a more in-depth understanding of the identified AF care pathways (quantitative component). The qualitative component comprised of focus group discussions (FGDs) and the quantitative component was longitudinal and descriptive in design. The inclusion criteria consisted of adults aged 18 years or older who spoke Brazilian Portuguese and with a diagnosis of AF or an arrhythmia likely to be AF who receive AF care from any of the included healthcare facilities. Patients were excluded if they had any hearing or cognitive impairment or if their home address was outside of São Paulo. Patients were first identified from primary and secondary electronic healthcare records (updated at every patient visit and includes all current patients receiving care from the included facilities) then phoned for an invitation to participate in the quantitative component of the study. Of the patients that were phoned, 20 were purposively chosen based on sex, age and sociodemographic status and subsequently invited to also take part in the qualitative component.

### Setting

**Fig 1:**
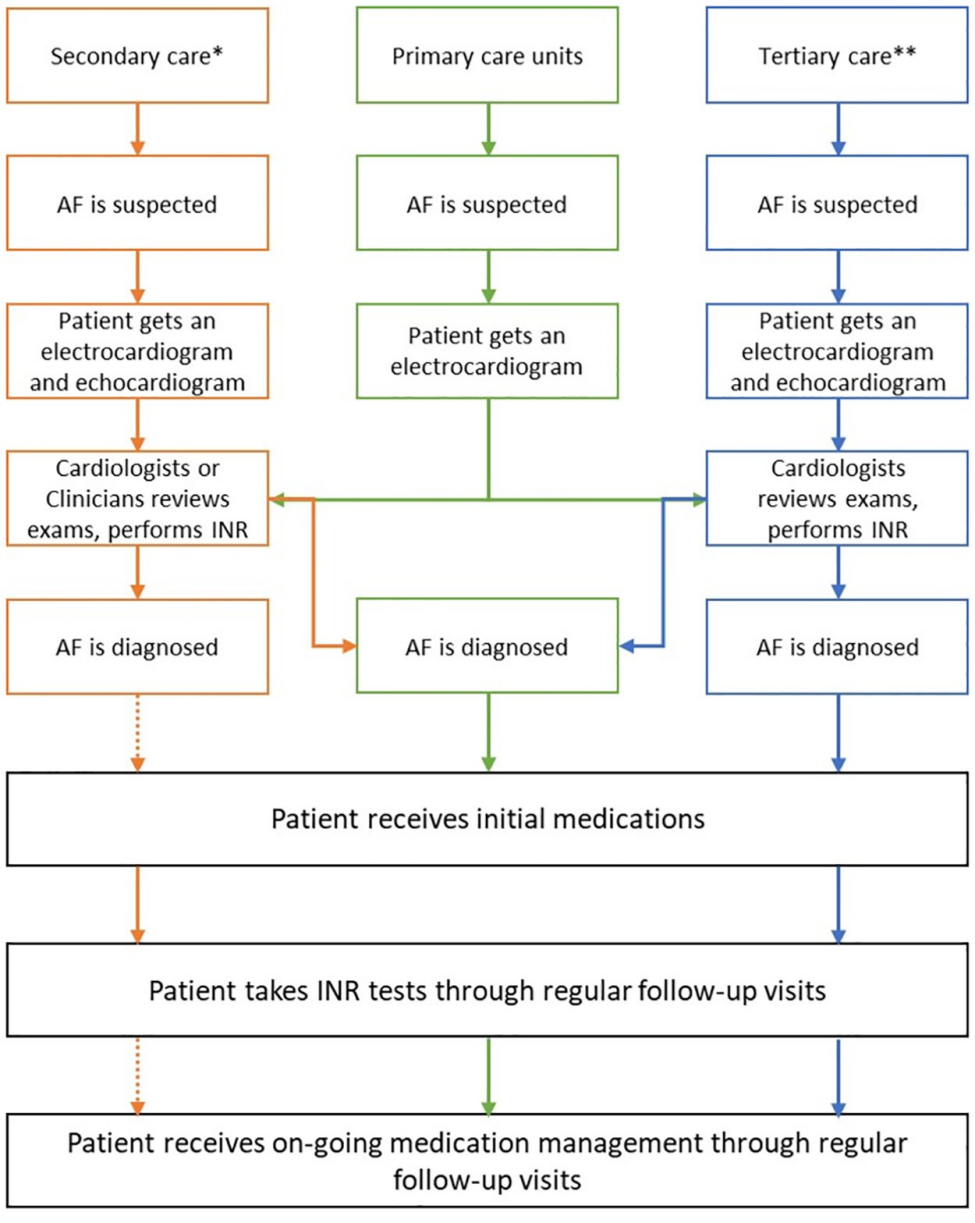
Possible pathways of care for atrial fibrillation (AF) in Brazil. * In Butantan, secondary care includes the specialised cardiology unit (Peri-Peri) and the outpatient clinic at the Hospital Universitario from the Universidade de Sao Paulo. ** There are no tertiary hospitals in Butantan. Solid line refers to a pathway that is always or nearly always available in Butantan; dotted line refers to a pathway that is not always or rarely available.

The study was conducted in Butantã District of São Paulo, an economically-deprived urban district with more than 540,000 inhabitants [11]. Based on available resources and local infrastructure, AF may be diagnosed within primary, secondary or tertiary care facilities in Brazil (Fig 1). As per national [12] and international guidelines [13, 14], anyone suspected with AF should take an electrocardiogram (ECG). If AF is confirmed, the patient should be referred for specialised AF management care where they would be prescribed OACs, and rate-limiting or antiarrhythmic medications based on internationally recognised evidence-based guidelines and risk assessment tools [13]. If the patient is prescribed warfarin, they should be referred for monthly INR tests [13]. In HICs, novel OACs (NOACs) are increasingly used which reduce the need for monthly INR tests; however, in Brazil, and many other LMICs, warfarin is the only anticoagulant available free of charge and is therefore a commonly prescribed anticoagulant among AF patients [15].

There are no tertiary hospitals in the Butantãn area, but there are fifteen primary care units, three secondary care facilities which include a specialised cardiology clinic (Peri-Peri), a 120-bed municipal community hospital and a 258-bed community university hospital (Hospital Universitário of the Universidade de São Paulo; HU-USP). Patients were recruited from facilities linked with the University of São Paulo which included eleven primary care units, the Peri-Peri and the HU-USP. Only one cardiologist is available in the Butantãn area, at the Peri-Peri specialised cardiology clinic, though the HU-USP has clinicians qualified for reviewing ECG results and advising on diagnoses.

### Quantitative data collection

The development of baseline and follow-up questionnaires are described elsewhere [10]. Two trained nurses administered the questionnaires in person or by phone. Baseline data were completed between June 2019 and November 2020, prior to the FGDs. The follow-up questionnaire was completed by each participant at least two months following the baseline questionnaire for the purpose of capturing follow-up care received during the study period. Only data collectors had access to participants names and contact information for the purpose of contacting them at follow up.

During data collection, the COVID-19 pandemic emerged, with the first case in Brazil documented in February 2020 [16]. São Paulo, the most populated city in Brazil, was especially impacted regarding the number of cases and deaths [17]. The likely impact of the pandemic on the health system meant that data captured on AF follow-up care after the start of the pandemic would differ from data captured prior to the pandemic; thus, data collected during the pandemic may not reflect care in normal circumstances. In response, we adapted our data collection tools and study aims to understand how the pandemic impacted AF follow-up care and to ensure we present our findings within the context of the emergent pandemic. We developed an additional short questionnaire regarding the impact of COVID-19 on hospital/clinic visits, receiving medications and conducting INR tests. These questions were only asked to those who completed the follow-up questionnaire after the pandemic commenced.

### Qualitative data collection

FGDs were carried out with AF patients to identify the drivers for where they receive their AF diagnosis and follow-up care [18]. Topic guides were developed in collaboration with members of the National Institute for Health and Care Research (NIHR) Global Health Research Group on AF Management from the Universities of São Paulo, Birmingham and Liverpool [19]. The topic guides were translated into Brazilian Portuguese then back translated into English to check for accuracy. Questions were focused on how the patient received their AF diagnosis and their health-seeking behaviours before and after their diagnosis (i.e. how, why and where they received their diagnosis and follow-up care). The same trained nurses that collected the quantitative data attended and took notes during the FGDs. An assistant professor (ACG) and anthropologist (BP), both female, trained in qualitative methods conducted the FGDs; neither were previously known to any of the participants. All FGDs were held in a private, quiet room within the HU-USP, with participants sat around a round table.

### Sample size calculation

The sample calculation for the quantitative component used an assumption that 16% of AF patients would be diagnosed in primary care [20]. A minimum sample size of 205 was required to accurately estimate the proportion of AF patients diagnosed in primary care in an unknown population size with ±5% accuracy at the 95% confidence level (α = 0.05). We increased the sample size by 15% to account for loss to follow-up, resulting in a minimum sample size of 236. For the qualitative component, we aimed to reach saturation with three FGDs, with up to ten participants in each [21, 22].

### Analysis

SPSS 27.0 was used for the quantitative analyses. Descriptive statistics were used to present all questionnaire data. Continuous variables are presented as mean with standard deviation (±SD) and categorical variables with frequencies and proportions.

All FGDs were audio recorded and transcribed verbatim in Brazilian Portuguese then translated into English. We conducted a content analysis [23], reading and re-reading the full transcripts line-by-line to identify and code any data related to AF diagnosis and AF follow-up care. Codes were combined and grouped into sub-themes under the two overarching themes of diagnosis and follow-up care. Data were independently analysed by two researchers (TEG and ACG) to reduce confirmation bias. TEG is a research fellow in global health at the University of Birmingham with extensive qualitative experience; TEG was not present for any FGD and has had no relationship with the participants. ACG is a senior medical researcher and assistant professor working in the field of cardiovascular disease and other chronic conditions at the University of São Paulo; ACG is trained in qualitative methods and was present for all FGDs.

### Patient and public involvement

Patient partners were not involved in the design or conduct of this study.

### Ethics statement

Ethical approval was received in Brazil from the Committee of Ethics in Research of the Hospital Universitário from the Universidade of São Paulo and the National Commission of Ethics in Research (approval number 3.301.920). Written informed consent was provided from all included participants either through a signature, or thumbprint on paper if illiterate, or verbally over the phone.

## Results

**Fig 2:**
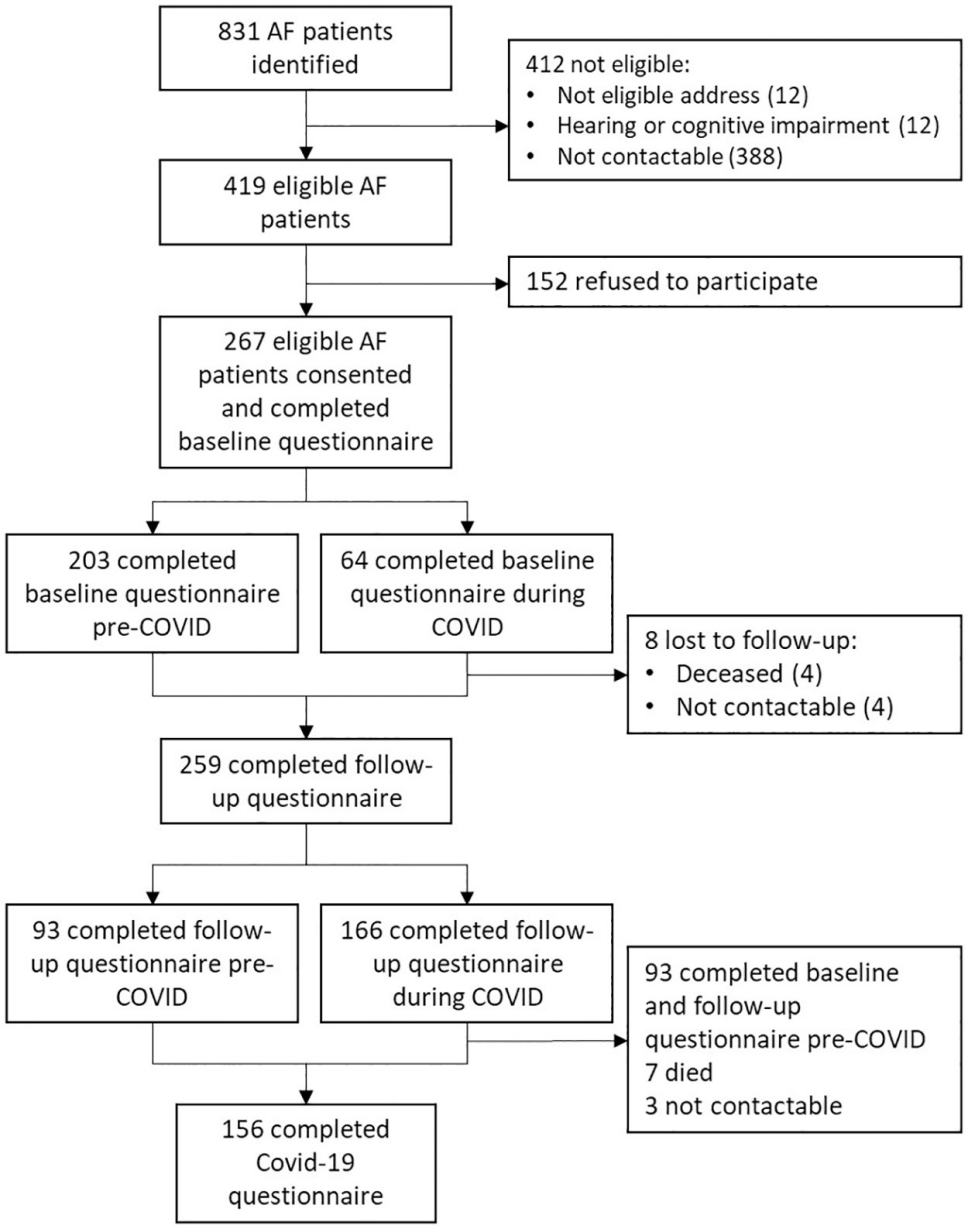
Study flow chart.

From electronic records, 831 AF patients were identified. Of these, 412 were excluded due to: not having an eligible address (n = 12) or having a hearing or cognitive impairment (n = 12). A further 388 patients were not reachable (Fig 2). Of the 419 eligible contacted patients, 152 declined to take part in the study (reasons were not obtained), leaving 267 patients eligible and consented for participation. Eight participants were lost to follow-up: four died and four could not be contacted despite a few attempts. Therefore 259 participants completed the follow-up questionnaire conducted on average 4.4 months following baseline. Of those who completed the follow-up questionnaire during the pandemic (n = 166), seven died and three could not be contacted for completion of the COVID-19 questionnaire; thus, data about care during the pandemic was collected from 156 participants. Participant characteristics are detailed in Table 1.

**Table 1:** Baseline characteristics of participants according to the time of data collection and in total; figures are presented as N (%) unless otherwise stated.

	Pre-COVID-19 (n = 203)	During-COVID-19 (n = 64)	Total sample (N = 267)
Age			
Mean (SD)	69.2 (11.5)	68.2 (10.4)	68.9 (11.3)
< 40	2 (1.0)	0 (0)	2 (0.7)
40 to 49	10 (4.9)	4 (6.3)	14 (5.2)
50 to 59	30 (14.8)	9 (14.1)	39 (14.6)
60 to 69	45 (22.2)	22 (34.4)	67 (25.1)
70 to 79	78 (38.4)	19 (29.7)	97 (36.3)
80 +	38 (18.7)	10 (15.6)	48 (18.0)
Gender			
Female	97 (47.8)	34 (53.1)	131 (49.1)
Male	106 (52.2)	30 (46.9)	136 (50.9)
Marital status			
Single	18 (8.9)	8 (12.5)	26 (9.7)
Married	107 (52.7)	33 (51.6)	140 (52.4)
Living with partner	14 (6.9)	6 (9.4)	20 (7.5)
Divorced	18 (8.9)	6 (9.4)	24 (9.0)
Widowed	42 (20.7)	9 (14.1)	51 (19.1)
Missing / unknown	4 (2.0)	2 (3.1)	6 (2.2)
Ethnicity			
White	104 (51.2)	30 (46.9)	134 (50.2)
Black	24 (11.8)	7 (10.9)	31 (11.6)
Mixed race	58 (28.6)	18 (28.1)	76 (28.5)
Other	4 (2.0)	2 (3.1)	6 (2.2)
Missing / unknown	13 (6.4)	7 (10.9)	20 (7.5)
Education			
Did not complete primary school	68 (33.5)	18 (28.1)	86 (32.2)
Completed primary school	74 (36.5)	17 (26.6)	91 (34.1)
Completed secondary education	34 (16.7)	19 (29.7)	53 (19.9)
Holds undergraduate degree	19 (9.4)	6 (9.4)	25 (9.4)
Holds postgraduate degree	1 (0.5)	2 (3.1)	3 (1.1)
Missing / unknown	7 (3.4)	2 (3.1)	9 (3.4)
Literacy			
Illiterate	14 (6.9)	4 (6.3)	18 (6.7)
Literate	189 (93.1)	60 (93.8)	249 (93.3)
Employment status			
Employed	34 (16.7)	10 (15.6)	44 (16.5)
Retired	145 (71.4)	37 (57.8)	182 (68.2)
Housewife	8 (3.9)	8 (12.5)	16 (6.0)
Student	1 (0.5)	0 (0)	1 (0.4)
Unable to work	15 (7.4)	5 (7.8)	20 (7.5)
Cannot find suitable job	0 (0)	3 (4.7)	3 (1.1)
Does not want to work	0 (0)	0 (0)	0 (0)
Other	0 (0)	1 (1.6)	1 (0.4)
Travel time to clinic			
Mean, minutes (SD)	57.1 (35.7)	45.2 (28.2)	54.2 (34.3)
Less than 30 minutes	40 (19.7)	13 (20.3)	53 (19.9)
Between 30 minutes and 1 hour	94 (46.3)	39 (60.9)	133 (49.8)
Between 1 and 2 hours	64 (31.5)	12 (18.8)	76 (28.5)
More than 2 hours	3 (1.5)	0 (0)	3 (1.1)
Missing / unknown	2 (1.0)	0 (0)	2 (0.7)
Mode of travel to clinic			
Walk	7 (3.4)	3 (4.7)	10 (3.7)
Bus or other public transit	122 (60.1)	27 (42.2)	149 (55.8)
Taxi	34 (16.7)	19 (29.7)	53 (19.9)
Family-owned vehicle	38 (18.7)	15 (23.4)	53 (19.9)
Other	1 (0.5)	0 (0)	1 (0.4)
Missing / unknown	1 (0.5)	0 (0)	1 (0.4)
Other adults living in the home			
0–2	134 (66.0)	44 (68.8)	178 (66.7)
3–5	66 (32.5)	20 (31.3)	86 (32.2)
More than 5	2 (1.0)	0 (0)	2 (0.7)
Missing / unknown	1 (0.5)	0 (0)	1 (0.4)
Live children (<18) living in the home			
0–2	192 (94.6)	59 (92.2)	251 (94.0)
3–5	1 (0.5)	0 (0)	1 (0.4)
More than 5	0 (0)	0 (0)	0 (0)
Missing / unknown	10 (4.9)	5 (7.8)	15 (5.6)
Number of rooms in the home			
1–2	7 (3.4)	2 (3.1)	9 (3.4)
3–4	119 (58.6)	44 (68.8)	163 (61.0)
More than 4	70 (34.5)	13 (20.3)	83 (31.1)
Missing / unknown	7 (3.4)	5 (7.8)	12 (4.5)
Comorbidities^a^			
Congestive heart failure	148 (72.9)	36 (56.3)	184 (68.9)
Hypertension/high blood pressure	172 (84.7)	54 (84.4)	226 (84.6)
Blood clot	60 (29.6)	20 (32.3)	80 (30.0)
Peripheral vascular disease	51 (25.1)	21 (32.8)	72 (27.0)
Hyperthyroidism	26 (12.8)	9 (14.1)	35 (13.1)
Long-term lung problems	20 (9.8)	3 (4.7)	23 (8.6)
Long-term kidney problems	28 (13.8)	2 (3.1)	30 (11.2)
Other (diabetes, Chagas, cancer, catarata)	20 (9.9)	3 (4.7)	23 (8.6)

^a^ Participants could choose more than one option; the percentages may not add up to 100%.

Twenty patients were invited to participate in the FGDs; however, three declined the invitation. We conducted three FGDs with the remaining 17 patients in groups of seven, five and five. There were eight participants over the age of 65, nine were female and 12 were married. FGDs were balanced in terms of age and sex but slightly differed in marital status, with no single participants included in the second FGD and no widowed participants in the third FGD.

### AF diagnosis

A quarter of participants (25%; 68/267) received their AF diagnosis in primary care units. Many participants received their AF diagnosis in a secondary or tertiary care facility (65%; 174/267), most of which occurred in an emergency (34%; 59/174) or inpatient (49%; 85/174) department. Eight percent (22/267) received their diagnosis in a private facility and 1% (2/267) were diagnosed by a traditional healthcare provider. One participant (<1%) was unsure where they were diagnosed.

### Follow-up care before and during the COVID-19 pandemic

Nearly all participants (93%; 242/259) said they had seen a healthcare professional about AF since baseline; with 204 (74%) providing details of where they visited and how often they had a healthcare visit. Of the 204 subjects, all but one participant visited the AF or INR clinic for follow-up care; additionally, 21–31% also visited their primary care unit and 3–8% visited private care facilities during the same period for AF related care.

Eighty-five participants had a at least one visit to a healthcare facility for AF care before the pandemic began (418 total visits, 1.5 average visits per month) and 119 participants had a healthcare visit for AF care during the pandemic (356 total visits, 0.7 average visits per month). Of the participants that said their healthcare visits for AF care were impacted by the pandemic (52%; 81/156), 83% (67/81) indeed stated they had fewer visits than usual; a further 12% (10/81) said they had to visit a different healthcare facility during the pandemic and 11% (9/81) said they had remote visits (e.g. on the phone) with the doctor or nurse.

Twenty-nine percent of participants (45/156) said the pandemic impacted their access to AF medication: 56% (25/45) said they were inaccessible at the pharmacy, 42% (19/45) had to get medication from a different facility than usual, 13% (6/45) could not obtain their medications during this time, 11% (5/45) said their medications were delayed and 4% (2/45) had their medications delivered to them.

Of 48 participants that said INR tests were impacted by the pandemic, 67% (32/48) had longer intervals between tests, 13% (6/48) had difficulties accessing a doctor for a test and 13% (6/48) of participants said they began to check their test results online. A large proportion of participants said their medical visits (63%; 51/81), medications (31%; 14/45) and INR tests (60%; 29/48) were affected by the pandemic because they feared getting COVID-19 or because they were isolating or quarantining. More than half also said the place where they usually go for care (53%; 43/81), medications (69%; 31/45) and INR tests (56%; 27/48) were closed or had reduced opening hours during the pandemic. A further 27% (22/81) of participants said local restrictions of curfews or lockdowns meant they were unable to visit their usual healthcare facility for AF care during the pandemic. Table 2 summarises the impacts of the pandemic on AF care.

**Table 2:** Impacts from the COVID-19 pandemic on AF follow-up care, as mentioned by participants.

Healthcare visits	AF medications	INR tests
Fewer overall visits Different healthcare facility than usual More virtual appointments	Not able to get prescriptions Prescriptions delayed Medication not available from pharmacy Different pick-up location Medications were delivered	Longer intervals between tests Tests were stopped Location of test changed Test results now online No access to tests

### Drivers for the identified AF care pathway

**Fig 3:**
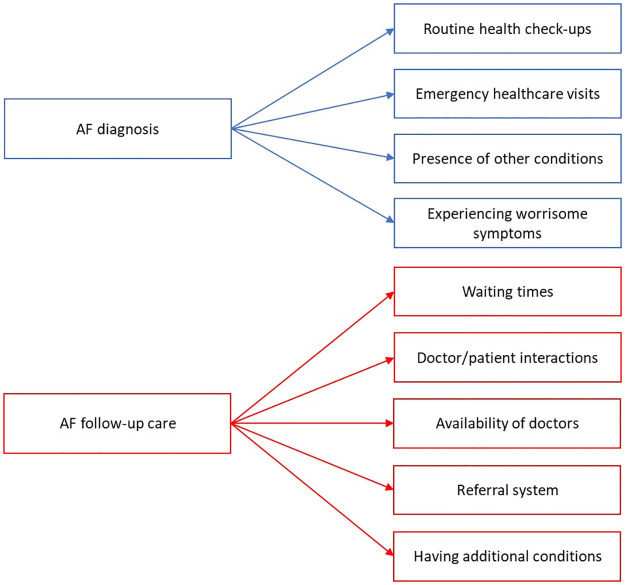
Coding tree from qualitative analysis.

From the qualitative data, we report the drivers for when and where AF patients received an AF diagnosis and follow-up care. Nine sub-themes in total were identified that describe the two main themes of ‘AF diagnosis’ and ‘AF follow-up care’, all of which were generated within the first two FGDs (i.e. saturation was met). The following four sub-themes describe the driving factors for place of ‘AF diagnosis’, our first theme (Fig 3):

1. Routine health check-ups: A few participants mentioned that AF was picked up through routine check-up examinations:

*“It’s an annual exam*. *Every year I visit the clinic to take an exam*. *Then I did an electrocardiogram and it recorded the arrhythmia*. *I was about 60 years old*, *more or less*.*”*

*(FGD 1*, *P3)*

2. Emergency healthcare visits: Many participants said AF was not identified until they had a healthcare emergency such as a myocardial infarction or thrombosis:

*“…until I had a heart attack*, *I didn’t know what I had in my heart*… *no doctor told me*. *Then after I had it … the doctor told me ’what you have is very serious’… He said [to my family] ‘she has severe heart failure and has arrhythmia’*.*”*

*(FGD 1*, *P1)*

3. Presence of other conditions: Other participants stated that their AF was picked up through the care they were receiving for other conditions:

*“I had physical therapy and my physiotherapist would look at how my oxygenation was*. *She came and said that my heart was beating 170*. *She asked me ‘are you normal*?*’*, *‘Is something happening*?*’ and I said ‘no’*. *A week later she started measuring again and was even more accelerated … I went to the cardiologist and … she found out that I had arrhythmia*, *but I never felt anything*.*”*

*(FGD 1*, *P5)*

4. Experiencing worrisome symptoms: Some participants said they experienced symptoms that prompted them to (or their family to assist that they) get checked by a doctor; however, many participants initially ignored their symptoms due to wrongly associating them with smoking or other conditions they had:

*“Then I started walking up a street and I started to get very tired*. *I was taking a shower and it started to give my head a bursting pain*. *I told my wife and we went straight to the doctor … he said*, *‘I have to admit to you now*, *it’s a bad situation*.*’*.*"*

*(FGD 2*, *P2)*

*“The slope in my house is really steep*. *To go up*, *wow*, *it was a tiring thing*. *But then I said ‘Oh*, *this is the result from my asthma and bronchitis’*.*”*

*(FGD 2*, *P5)*

Four sub-themes were identified regarding the driving factors for where patients go for ‘AF follow-up care’, our second theme (Fig 3):

1. Waiting times: Participants often mentioned the long waiting times to see a doctor and to receive their INR results; thus, many chose private care over public due to the long waiting times in the latter, but acknowledged that they regularly use both private and public services:

*“But it takes a while*, *you don’t ask today and tomorrow you’re at the cardiologist*. *If it takes a long time*, *and I’m more worried or with a lot of doubts*, *I pay a private person because it’s quick and it clarifies my doubts*.*”*

*(FGD 2*, *P4)*

*“When it takes longer*, *I pay privately and take my INR exams*.*”*

*(FGD 3*, *P2)*

2. Doctor and patient interactions: Many participants commented on the importance of their interactions with doctors and how this affected their attendance for and attitude toward follow-up care:

*“I go every 6 months to my PCU [primary care unit]*. *One day I arrived and they told me that I would be treated with … a doctor*, *she was very rude*, *I didn’t like her*, *then I complained*, *they told me to change the days from Tuesdays and Thursday for Friday*. *When I got there*, *the doctor was the best doctor I’ve ever met*.*”*

*(FGD 3*, *P2)*

3. Health system factors: Participants spoke about the availability and access to cardiologists where primary care physicians are gatekeepers. To see a cardiologist for any concerns or questions between INR tests, participants said they must first visit the primary care unit to ask for a referral; thus, they are required to visit two separate facilities at minimum, adding additional burden to the patient, healthcare system and a delay in care:

*“When I went to the PCU*, *I asked [about prescriptions]*, *but the doctor didn’t answer me*, *only saying that it is ideal for me to see the cardiologist*.*”*

*(FGD 2*, *P1)*

*“I get to PCUs; they don’t have a doctor and from there they send me to another place*. *You are on hold*.*”*

*(FGD 2*, *P2)*

4. Additional conditions: Many participants had comorbidities. Whilst having multiple conditions often means attending various healthcare appointments, some said they received care for AF at the same place and time as they received care for additional conditions:

*“Now they’re going to transfer me to InCor [tertiary care facility outside Butantan] because here I have both things*. *So*, *there they treat me for both things*.*”*

*(FGD 3*, *P1)*

## Discussion

In this mixed-methods study investigating the existing pathways of care, their drivers and the impact of COVID-19 on service delivery to AF patients in Brazil, we identified three critical findings which have patient outcome, resource and health systems implications relevant to Brazil and other LMICs. First, a large proportion of participants received their AF diagnosis as inpatients or in the emergency department after they experienced a medical emergency or severe symptoms. Second, many participants visited two or more types of healthcare facilities to receive follow-up care, which was driven by inadequate waiting times and other health system factors. Lastly, the COVID-19 pandemic impacted all aspects of follow-up care; however, certain strategies used to maintain care during the pandemic provide insights on how AF care could be optimised during and beyond future crises.

Only a quarter of the participants in our study were diagnosed in primary care which led to most diagnoses being made following a major health event such as a stroke. Thus, our study findings are potentially underreporting the burden of late diagnosis as AF patients who may have died from myocardial infarction or stoke would not appear in our study. To reduce AF-related morbidity and mortality, primary care units should be well trained and prepared to recognise AF-related symptoms and conduct appropriate investigations. However, we know from previous research [15] that AF training is lacking in primary care units in São Paulo which can result in AF symptoms being ignored or misdiagnosed, thus depriving patients of life-saving medication. This was found in our other two project sites in Sri Lanka and China [24], confirming that poor primary care training in recognition, diagnosis and management of AF is likely a common problem in many LMICs. Avoiding stroke with anticoagulation is a key pillar of evidence-based optimal management of AF (ABC pathway) [6], but improved clinician awareness, use of clinical risk stratification [13], skills to conduct initial investigation and knowledge of treatment options by primary care staff is vital for enabling this to occur. Improved AF training could also improve primary care physicians’ confidence and ability to answer patients’ queries in between INR tests to avoid unnecessary referrals and additional healthcare visits to secondary care.

For primary care staff to effectively monitor and detect AF, patients must first visit their primary care doctors early in their symptomatology, but as our qualitative data suggests, many AF patients do not seek healthcare until symptoms have become debilitating, or a medical crisis has occurred, driven in part by poor patient health-literacy and mistakenly associating their symptoms with other existing conditions. A qualitative study corroborates these findings, with evidence that late AF diagnosis is in part due to a lack of patient awareness about AF in Canada [25], indicating the relevancy of our findings to HICs as well. Future research should investigate how public knowledge could be improved on how AF is a relatively common, easily diagnosed and potentially catastrophic condition that can be prevented, and how healthcare seeking behaviour change can be achieved to increase rates of diagnosis and preventive care before a medical emergency occurs. Educational methods for self-management of AF has been studied [26] and could be adapted and applied in LMICs, including but not limited to low-literacy picture-based educational materials, online educational materials, nurse-led face-to-face educational sessions, scheduled telephone follow-ups, and high-intensity, multidisciplinary education sessions [26].

An early diagnosis is not beneficial without adequate follow-up care. AF patients in our study often had chronic comorbidities, and even for AF care many received follow-up care at two or more different facilities. Management of all chronic conditions requires continuity of care to ensure multiple medications, which often interact or can have side-effects, and ameliorating life-style behaviours are systematically advised to patients and their care managed in a coherent integrated way across time [27]. This is a challenge in HICs and LMICs. Our evidence that patients seek multiple providers for AF care, indicates sub-optimal continuity of care, and puts into question the quality of healthcare they receive, since for example disruption to continuity of care, particularly for patients on warfarin can increase the risk of stroke and other morbidities; thus, premature mortality can occur [28, 29]. Additionally, such multiple visits are inefficient and can be a burden and costly to patients, the health system and society [28]. Providing respectful and compassionate follow-up care (e.g. ensure positive doctor/patient interactions) which should be easily accessible (e.g. a reduction in waiting times and unnecessary referrals) are known quality indicators for patient-centred care [30, 31] and can positively influence continuity of care, along with patients’ health-seeking behaviours and adherence to the long-term follow up care required for AF [25].

The COVID-19 pandemic impacted AF follow-up care. Whilst most participants maintained access and adherence to medication and INR tests during the pandemic, disruptions to receiving medications and longer intervals between INR tests were reported. This was in part due to fear of being infected by COVID-19; thus, some participants avoided going for tests. As 56% of our participants used public transportation to access healthcare facilities, the fear of COVID-19 was likely intensified by the possibility of being exposed to the virus through use of public transport. Additionally, facility closures and reduced hours left many patients with the only option to buy their medications from private pharmacies; this was also reflected in a Brazilian study that investigated the impact of COVID-19 on the management of other NCDs [32]. Worldwide, patients with AF and other NCDs found that creative service delivery virtually via phone or internet and delivery of medication during the pandemic was desirable [33, 34]. Delivering care at or close to patients’ homes could reduce the need for patients to take public transportation and purchase medications from private facilities during a pandemic or natural disasters and emergencies. Additionally, increasing the access to NOACs in LMICs which require less follow-up than warfarin whilst providing similar efficacy [35] should be considered given the risk and barriers for continuity of care during and beyond major healthcare disruptions. Such solutions could save heath system and patients’ time and money by reducing the number of visits to healthcare facilities, and could be beneficial for patients with disabilities or reduced mobility, particularly in LMICs where facilities can often be geographically far and difficult to get to.

### Strengths and limitations

We presented data on the AF care pathway pre- and during the COVID-19 pandemic and the drivers behind the identified pathway in Brazil. A key strength of our study is the mixed-methods design, enabling our ability to identify where patients go and why. Additionally, we included all except two public healthcare facilities located in the Butantãn district of São Paulo; thus, we were able to capture the pathway of AF care using a representative sample of AF patients receiving governmental care across the region. Limitations to mention include the long duration since AF diagnosis; on average, participants were diagnosed with AF 11 years prior to data collection that may have introduced some recall bias, although most participants clearly remembered when they were first diagnosed and other questions were asked about relatively recent events. Whilst our findings may not fully reflect newly diagnosed cases, we did have newly diagnosed patients who demonstrated the same issues, and the health system has not undergone any major changes in the last decade; therefore, we expect our results to be currently relevant. This study recruited patients with a confirmed diagnosis of AF currently receiving follow-up care; therefore, AF patients not receiving care and patients receiving mostly private care were likely missed from recruitment. As mentioned elsewhere [10], the large proportion of eligible patients that were not contactable and did not want to take part could have introduced selection bias and our results should be interpreted with caution. It is possible that the unreachable patients represent a more hard-to-reach population who experience additional or different barriers to accessing healthcare. Although our participants represented both urban and rural inhabitants from an impoverished area of Brazil, the public healthcare system in São Paulo is better established than some other macro regions of Brazil and therefore may not be generalisable and transferable to areas with fewer facilities where the situation may be less favourable for AF patients.

## Conclusion

We demonstrate that in our setting few AF diagnoses occur in primary care, resulting in late diagnosis, delayed medications, and costly medical emergencies including stroke and myocardial infarction. Additionally, patients visit more than one facility for follow-up care which can disrupt continuity of care and be a burden to patients and the health system; this was driven by a complex web of causes including long waiting times and an inadequate referral system. These findings all point to a need to improve primary care capabilities for diagnosis and management of AF and patient-centred care in general, as well as better public and patient education aiming beyond improved knowledge to create behaviour change. The COVID-19 pandemic also proved to be a disruption to all aspects of continuity of AF care, but creative service delivery can be formulated and incorporated in disaster management plans to assist with better management of AF and other NCDs. Expanding pandemic-related innovations on delivering care closer to patients’ homes and improving access to NOACs could make follow-up care more efficient and consequently avoid AF-related morbidity and premature mortality beyond epidemics and times of crises. Lessons from Brazil seem to reflect reports from other LMICs and some HICs, thus pointing to a need for a global impetus to review services for AF, the most common cardiovascular arrhythmia with life-threatening and disabling consequences if diagnosed late or left untreated.

## Supporting information

S1 Checklist STROBE statement—Checklist of items that should be included in reports of *cross-sectional studies*. (DOCX)
